# Humidification Performance of Heat and Moisture Exchangers for Pediatric Use

**DOI:** 10.1155/2012/439267

**Published:** 2012-01-18

**Authors:** Yusuke Chikata, Chihiro Sumida, Jun Oto, Hideaki Imanaka, Masaji Nishimura

**Affiliations:** ^1^The University of Tokushima Graduate School, 3-18-15 Kuramoto-cho, Tokushima City, Tokushima 770-8503, Japan; ^2^Faculty of Medicine, The University of Tokushima, 3-18-15 Kuramoto-cho, Tokushima City, Tokushima 770-8503, Japan; ^3^Department of Emergency and Critical Care Medicine, The University of Tokushima Graduate School, 3-18-15 Kuramoto-cho, Tokushima City, Tokushima 770-8503, Japan; ^4^Department of Emergency and Disaster Medicine, Tokushima University Hospital, 2-50-1, Kuramoto-cho, Tokushima City, Tokushima 770-8503, Japan

## Abstract

*Background*. While heat and moisture exchangers (HMEs) have been increasingly used for humidification during mechanical ventilation, the efficacy of pediatric HMEs has not yet been fully evaluated. *Methods*. We tested ten pediatric HMEs when mechanically ventilating a model lung at respiratory rates of 20 and 30 breaths/min and pressure control of 10, 15, and 20 cmH_2_O. The expiratory gas passed through a heated humidifier. We created two rates of leakage: 3.2 L/min (small) and 5.1 L/min (large) when pressure was 10 cmH_2_O. We measured absolute humidity (AH) at the Y-piece. *Results*. Without leakage, eight of ten HMEs maintained AH at more than 30 mg/L. With the small leak, AH decreased below 30 mg/L (26.6 to 29.5 mg/L), decreasing further (19.7 to 27.3 mg/L) with the large leak. Respiratory rate and pressure control level did not affect AH values. *Conclusions*. Pediatric HMEs provide adequate humidification performance when leakage is absent.

## 1. Introduction

In spontaneous breathing subjects, inspiratory gases are heated and humidified in the nasal cavity and pharynx; by the time, they reach the second bronchial bifurcation, temperature reaches 37°C, and absolute humidity reaches (AH) 44 mg/L [1]. In mechanically ventilated patients, because artificial airways bypass this natural gas conditioning, administered gases require heating and humidification. Inadequate humidification induces inspissations of airway secretions, destruction of airway epithelium, and hypothermia [2]. The incidence of endotracheal tube occlusion was reported between 0.8% and 2.6% in pediatric patients [3, 4]. The American Association for Respiratory Care (AARC) recommends that inspiratory gases should reach a temperature of more than 30°C and AH of more than 30 mg/L [2].

Heat and moisture exchangers (HMEs) and heated humidifiers (active humidification) are commonly employed to warm and humidify medical gases. While HMEs are widely used during the mechanical ventilation of adults [5], their performance varies depending on model [6] and is affected by location, tidal volume, and minute volume [7, 8]. For pediatric patients, because HME adds significant dead space and airway resistance to the circuit, and because there is a high risk of inadequate humidification owing to leakage around the endotracheal tube (ETT), long-term use of HMEs is generally avoided. Few studies, however, have reported how ventilator settings and ETT leakage influence the humidification performance of HMEs used for pediatric patients.

Using a pediatric model lung, we carried out a bench study to evaluate the effects of ventilator settings and leakage volume on the humidification performance of pediatric HMEs.

## 2. Materials and Methods

**Figure 1:**
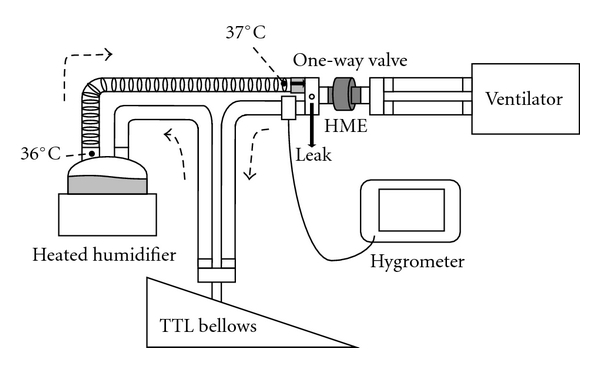
Experimental setup. In a circuit including a heated humidifier, a limb with a heating wire, and a one-way valve connector, a ventilator delivered medical gases, through the HME being tested, to the bellows. The gases were warmed to 36°C at the chamber outlet of the heated humidifier and to 37°C at the end of the limb. Two levels of leakage were created: 3.2 L/min (small) and 5.1 L/min (large) at airway pressure of 10 cmH_2_O. HME: heat and moisture exchanger.

Ten different models of HME were investigated (Table 1). All HMEs were designed for pediatric use, and not to be recommended for use in adult patient. Using a pediatric model lung (TTL model 1601, Michigan Instruments, Grand Rapids, MI) and a heated humidifier (MR730, Fisher & Paykel, Auckland, New Zealand), we simulated real-life ventilatory conditions (Figure 1). A ventilator (Puritan-Bennett 840: Covidien, Carlsbad, CA) delivered medical gases through each tested HME to the TTL bellows, the heated humidifier, a limb with the heating wire, and a one-way valve connector to avoid contamination with expired gases. The compliance of the TTL bellows was set at 0.01 L/cmH_2_O. By inserting connectors with calibrated holes, we created two levels of leakage: 3.2 L/min (small) and 5.1 L/min (large) under airway pressure of 10 cmH_2_O. Ventilatory settings were assist-control mode, respiratory rates of 20 and 30 breaths/min, pressure control of 10, 15, and 20 cmH_2_O, inspiratory time of 0.8 s, positive end-expiratory pressure of 3 cmH_2_O, and F_I_O_2_ of 0.21. At the airway opening of the simulated respiratory system, we measured AH, RH, and temperature using a rapid response capacitance-type moisture sensor (Moiscope, Senko Medical, Tokyo, Japan) described previously [7, 9]. Ambient relative humidity (RH) was maintained between 51% and 61%, and ambient temperature at 23.8°C to 24.8°C. The inspired gases were warmed to 36°C at the chamber outlet of the heated humidifier and to 37°C at the end of the limb. After confirming the values of RH and temperature had stabilized, we recorded the values for 5 min. A stabilization of these values was defined as when the RH varied within 2% and the temperature varied within 0.3°C for 5 min described previously [9, 10]. At each change of ventilator settings, before recording data, we allowed at least 10 min and confirmed stabilization. All signals from the hygrometer were led to an analog/digital converter and recorded at 50 Hz/channel on computer using data acquisition software (WINDAQ, Dataq Instruments, Akron, OH).

The resistance of all HMEs was measured with a pressure sensor (TP-603T, ±50 cmH_2_O, Nihon-Koden, Tokyo, Japan) placed between a flow generator and the HMEs. Thirty liters of flow was created by the flow generator and measured with a pneumotachometer (model 4700, 0–160 L/min, Hans-Rudolph Inc., Kansas City, MO, USA) connected to a differential pressure transducer (TP-602T, ±5 cmH_2_O, Nihon-Koden, Tokyo, Japan). Flow and pressure signals were amplified, digitized, and recorded at 100 Hz per signal using data acquisition software (WINDAQ, Dataq Instruments, Akron, OH, USA).

 Results are expressed as mean ± SD. Analysis of variance was performed using repeated measures ANOVA, and Scheffé's test was performed as a post hoc test for multiple comparison. For each pediatric HME we tested, we examined the relationship between dead space and obtained AH values. The relationship between variables was assessed using Spearman's rho (rs). We also calculated the AH delivered/dead space (volume of HME) as an index of humidification efficiency. Comparison between our measured data and manufacturer data for resistance was obtained by Wilcoxon signed-rank test. All statistical tests were two sided, and a *P* value < 0.05 was considered statistically significant. All statistical analysis was performed using commercial software (SPSS 11.01, SPSS, Chicago, IL).

## 3. Results

**Figure 2:**
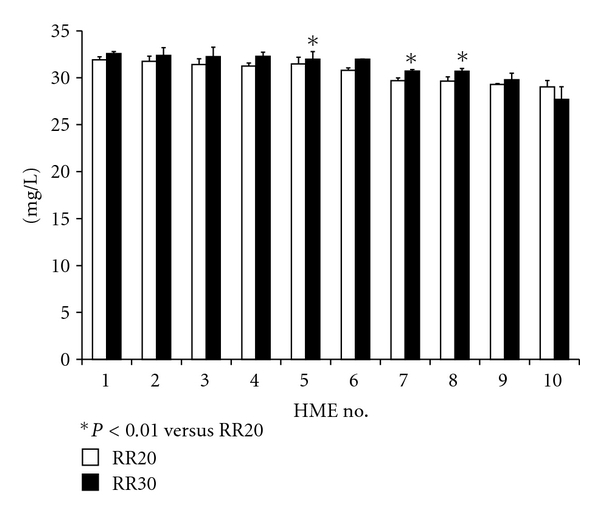
Effect of respiratory rate on absolute humidity in the absence of leakage. This figure shows the pooled results for pressure control settings of 10, 15, and 20 cmH_2_O without leakage. With three HMEs, increasing the respiratory rate statistically significantly increased absolute humidity. Mean ± SD; *P < 0.01 versus respiratory rate 20 breaths/min. HME: heat and moisture exchanger. RR: respiratory rate (breaths/min).

**Figure 3:**
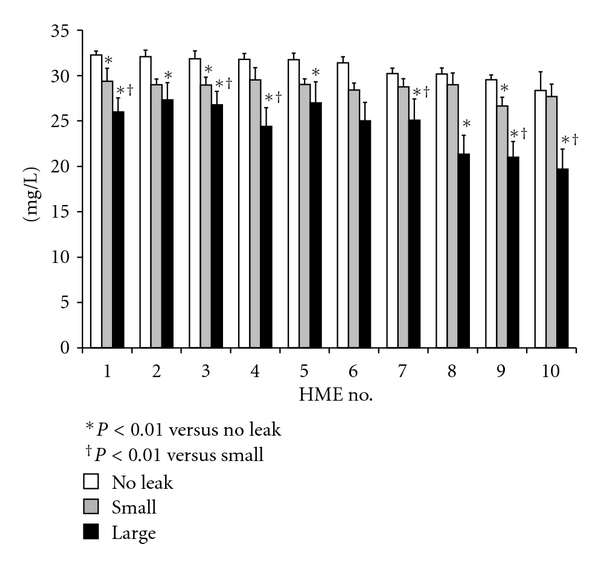
Effect of leakage on absolute humidity. This figure shows the pooled results for respiratory rates of 20 and 30 breaths/min, and with pressure control set at 10, 15, and 20 cmH_2_O. HME: heat and moisture exchanger. Mean ± SD; *P < 0.01 versus no leakage; ^†^ *P* < 0.01 versus small leak.

**Figure 4:**
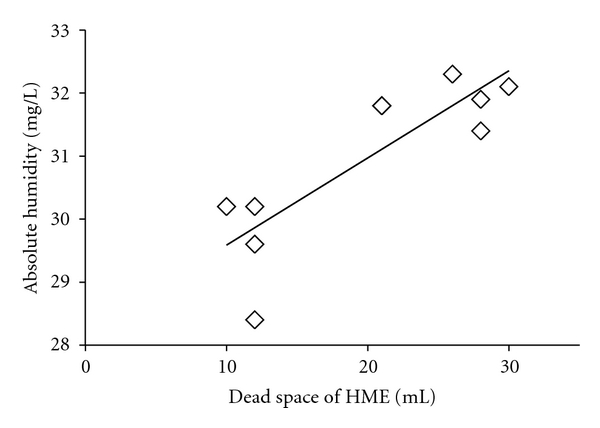
Relationship between absolute humidity and dead space in each HME. There was a statistically significant correlation between HME dead space and AH values (rs = 0.85, *P* = 0.018). HME: heat and moisture exchanger.

AH of expired gas at the end of the limb was about 36.5 ± 0.6 mgH_2_O/L, RH was 100%, and temperature was 33.4 ± 0.4°C. Without leakage, recorded AH varied (28.4 to 32.4 mg/L) among the tested HMEs, the average being 30.9 ± 1.5 mg/L. Eight of the ten HMEs maintained AH at more than 30 mg/L. The levels of pressure control (tidal volume) did not statistically significantly affect AH (data are not shown). As the respiratory rate increased, AH increased in three HMEs, but the percentage difference between respiratory settings of 20 and 30 breaths/min always remained less than 3%; changes in AH values with the other seven HMEs were negligible (Figure 2). With the small leak, 21.8% ± 5.6% of tidal volume was lost, and with the large leak, 44.2% ± 10.3%. When there was leakage in the circuit, none of the HMEs were able to maintain AH above 30 mg/L. With greater leakage, AH decreased with all HMEs (Figure 3). When the leak was small, AH was 28.6 ± 1.3 mg/L (26.6 to 29.5 mg/L), a 7.5% loss (92.5% of AH without leakage). With the large leak, AH dropped by 22% to 24.4 ± 3.2 mg/L (19.7 to 27.3 mg/L) (78% of AH without leakage). The mean ratio of AH to dead space among ten HMEs was 1.8 ± 0.7 (range: 1.1–3.0). There was a significant correlation between dead space of HME and AH values (rs = 0.85, *P* = 0.018) (Figure 4).

The average resistance of 10 HMEs was 4.3 ± 4.0 cmH_2_O/L/s, with a maximum of 13.6 cmH_2_O/L/s and a minimum of 0.6 cmH_2_O/L/s. The mean difference between our values and those of the manufacturers' reports was −1.0 ± 2.6 cmH_2_O/L/s (*P* = 0.27). Values were summarized in Table 1.

## 4. Discussion

We investigated the effects of leakage volume and ventilatory settings on humidification performance in ten HMEs for pediatric use. Without leakage, eight of the ten pediatric HMEs performed within the AARC guideline recommendation (AH min, 30 mg/L). When there was leakage, whether large or small, AH decreased below 30 mg/L in all pediatric HMEs. 

After investigating adult HMEs and antibacterial filters, Lellouche et al. found that 18 of 48 (38%) units were able to maintain AH at 30 mg/L or more, and the highest level of AH attained 31.9 mg/L [6]. There have been few reports, however, on the humidification performance of HMEs for pediatric use. Meanwhile, after investigating humidification with neonate patients, Fassassi et al. reported that use of an HME provided less AH (32.4 ± 2.8 mg/L) than a heated humidifier [11]; Schiffmann et al. reported that both HMEs (34 ± 2.6 mg/L) and heated humidifiers (33.8 ± 2.9 mg/L) provided enough AH in mechanically ventilated neonates and infants to meet the AARC guideline recommendation [12]. In these reports, however, because the protocol measured humidity between the tracheal tubes and humidifying device, it is likely that contamination with fully humidified expiratory gas increased the recorded AH values. This conjecture is supported by the findings of Luchetti et al., who showed inspiratory gas temperatures below 30°C and AH of less than 28 mg/L in all HMEs used with pediatric patients (3–33 kg) [13]. In our study, to accurately measure inspiratory gas humidity, as in the Luchetti et al. study, we inserted a one-way valve to prevent the mingling of inspiratory and expiratory gases. 

The HMEs we tested were able to maintain AH at a level in the range from 28.4 to 32.4 mg/L. AH ranging from 30 to 25 mg/L is usually considered reluctantly acceptable but not desirable, while AH of less than 25 mg/L exceeds threshold and should be avoided during mechanical ventilation [6]. With a large leak in the circuit, AH fell below 25 mg/L in five of the ten pediatric HMEs we tested. 

Comparing manufacturer data with actual performance, Lellouche et al. have reported that different HMEs have different performance, some performing so poorly that they should not be used [6]. While the reasons for differences were not clear in the ten units we tested, we did observe a tendency for HMEs with less dead space to provide less AH (Figure 4). It may be that the extra internal volume of HMEs with more dead space is related to the ability to maintain higher levels of humidity. When the respiratory rate increased, the AH increased with three HMEs and did not change with the remaining seven devices. When, from 10 cmH_2_O, the pressure control setting was increased to 20 cmH_2_O, AH tended to fall by approximately 3% although this decrease was not statistically significant. HMEs with little dead space may struggle to humidify gases flowing in large tidal volumes. Lucato et al. have reported that HMEs were more efficient when used with low tidal volume ventilation [8].

For mechanically ventilated adults with humidification provided with an HME, when air leakage increases, inspiratory gas humidification decreases [14, 15]. Consequently, AARC guidelines recommend not using an HME when leakage volume exceeds 30% of inspiratory tidal volume [2]. However, the humidification performance of pediatric HMEs has not been clarified. Luchetti et al. reported that AH was less than 30 mg/L when HMEs were used for pediatric patients with uncuffed ETTs [13]. Schiffmann et al. has also suggested, for neonate and pediatric patients, that HME humidification performance decreases when leakage volume exceeds 15% [12]. The relationship between leakage volumes and AH, however, has not been examined. As far as we know, this is the first report to evaluate, for pediatric patients, the effect of leakage volume on AH in inspiratory gas. Although, in the absence of leakage, most of the HMEs we tested could maintain AH at more than 30 mg/L, we found that when there was leakage, with all HMEs, AH decreased below 30 mg/L (Figure 3).

This study has several limitations. Naturally, because it is based on a model-lung simulation, the findings do not directly correspond to clinical situations. Moreover, our observation period was relatively short; the performance and safety of HMEs in prolonged mechanical ventilation remain to be proven. We simulated real-life ventilatory conditions using HH and AH of expired gas which were slightly higher than those of previous study [6]. This might lead to overestimation of inspired gas humidity in the present study.

In conclusion, without respiratory circuit leakage, the majority of the HMEs for pediatric use provided sufficient humidification. With leakage, however, humidification decreased and was not maintained at guideline values. If there is leakage around the tracheal tube, extra caution is advised when using an HME for humidification for pediatric patients.

## Figures and Tables

**Table 1:** Features of tested heat and humidifier exchangers.

No.	Device	Manufacturer	Recommended tidal volume (mL)	Measured resistance, cmH_2_O/L/s	Resistance from manufacturer's data, cmH_2_O/L/s	Type of HME	Dead space (mL)
(1)	Hygroboy	Tyco Healthcare	75–300	5.7	4.2	hygroscopic	26
(2)	HCH 5701	Vital signs	100–1,200	0.6	1.9	hygroscopic	30
(3)	Pharrma Mini	Pharma Systems	50–900	4.3	4.2	hydrophobic	28
(4)	HMEF Mini	GE Healthcare	60–500	3.5	3.0	hygroscopic	21
(5)	Servo Humidifier 161	Maquet	70–600	1.6	1.2	hygroscopic	21
(6)	Clear-Therm Mini	Intersurgical	75–200	3.3	3.4	mix	28
(7)	Humid-Vent 1	Hudson RCI	50–600	1.2	0.9	hygroscopic	10
(8)	Hygrovent Child	Medisize	50–250	13.6	6.0	hygroscopic	12
(9)	Thermovent 600	Smiths Medical	<600	1.2	2.9	hydrophobic	12
(10)	Vent Aid SK300CP	Fuji Medical	50–300	8.1	6.0	hygroscopic	12
